# Early HIV diagnosis through use of rapid diagnosis test (RDT) in the community and direct link to HIV care: a pilot project for vulnerable populations in Athens, Greece

**DOI:** 10.7448/IAS.17.4.19619

**Published:** 2014-11-02

**Authors:** Eleni Kakalou, Vasileios Papastamopoulos, Panagiotis Ioannidis, Kostas Papanikolaou, Ourania Georgiou, Athanasios Skoutelis

**Affiliations:** Infectious Diseases, Evangelismos General Hospital, Athens, Greece

## Abstract

**Introduction:**

An increase in the incidence of HIV new infections among intravenous drug users (IDUs) by 1500%, was noted in the center of Athens in 2011. Increasing problematic drug use, homelessness, health cuts amidst the economic crisis, have contributed to the epidemic. New cases doubled within a year, challenging the HIV care delivery system [1].

**Materials and Methods:**

A pilot project funded by the National Strategic Reference Framework (NSRF) 2007–2013 of the European Union (EU), was launched from August 2012 to March 2014. It was a partnership between the HIV Clinic of Evangelismos Hospital and the NGO PRAKSIS. The project is aimed at offering early diagnosis and comprehensive care to hard to reach populations. RDT diagnosis through mobile units, direct linkage to care, elimination of waiting times, flexibility, psychosocial support and link to harm reduction services for active IDUs were offered to the beneficiaries.

**Results:**

A total of 117 people enrolled in the program following HIV RDT offered by mobile units of the NGO PRAKSIS in community sites. Sixty-eight percent were IDUs, 12% were men who have sex with men (MSM) and 19.5% were heterosexuals. Men were 74.3% and women were 25.6%. Country born patients were 43.5% and non-country born patients were 56.4%. Nine people were HIV negative but needed post-exposure prophylaxis (PEP), treatment for Hepatitis C and one test was false positive. Two deaths occurred and six people were deported. Of the remaining 100 patients, 84 enrolled in the care program. Of those 77% (65/84) remain in care for three months after the end of the project. Care retention was 73.5% (39/53) for IDUs, 91% (10/11) for MSM, 80% (16/20) for heterosexuals, 73% (25/35) for country born and 82% (40/49) for non-country born individuals. Among those that remain in care, 77.7% (42/54) with <350 CD4 are on antiretroviral therapy (ART). Among those on ART >90% have undetectable viral load. Mean value of CD4 cells at enrollment was 298 cells/mm^3^. At follow up, three months after the end of the program, the mean value of CD4 cells was 464 cells/mm^3^.

**Conclusions:**

The project has proven the feasibility of a novel approach of active case finding in the community with direct link to care. Retention to care was satisfactory as most of those patients would not have been able to access care through the normal ART delivery mode of the Public Health System. However, more obstacles to care remain. Being homeless, poor nutrition, complicated access to harm reduction services, lack of One Stop Shop services and police operations in the city center impede further progress [2,3].
